# Idiopathic multifocal myocardial atrophy with fibrosis and fatty infiltration involving Purkinje fibres in a 13‐year‐old Arabian broodmare: Histopathological features

**DOI:** 10.1002/vms3.350

**Published:** 2020-09-17

**Authors:** Midori Goto Asakawa, Wasiq Mehmood, Mohammad Ali, Masa‐aki Oikawa

**Affiliations:** ^1^ Clinical and Anatomic Pathology Unit, Veterinary Specialists and Emergency Center Kawaguchi Japan; ^2^ Diagnostic and Research Laboratory Equine Veterinary Medical Center Doha Qatar

**Keywords:** Arabian horses, cardiac conduction system, cardiopathology, histopathology, Purkinje fibres, sudden death

## Abstract

Myocardial atrophy with fibrosis and fatty infiltration involving the cardiac conduction system is relatively unusual in horses. We herein report of such a case in a 13‐year‐old Arabian broodmare that had spontaneously died on a paddock. An autopsy revealed multifocal myocardial atrophy with concomitant fibrosis and fatty infiltration in both the ventricles and interventricular septum. The Purkinje fibres in the ventricles and interventricular septum were surrounded by thick fibrous or adipose tissues adjacent to atrophic myocardial cells. Myocardial fibrosis and fatty infiltration were likely secondary to myocardial atrophy, occurring as a pathological response triggered by the repair of muscular wall injury. However, there were no major vascular pathologies (e.g. atherosclerosis and arteriosclerosis); hence, the pathogenesis of myocardial atrophy was unclear. There was no evidence of myocardial atrophy - induced pathologies such as infarct, ischaemic lesions, myocardial degeneration, myocarditis and endocarditis. However, such an unusual histopathological pattern may be associated with rapid clinical deterioration and death.

## Graphical abstract

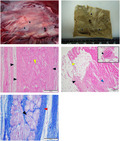

## 1 INTRODUCTION

Compared with what is known about the pathologies of equine organ systems such as the locomotor, gastrointestinal and nervous systems, data on pathologies of the equine cardiac system are limited. Most recent studies on cardiac pathologies have focused on racehorses, with special attention to sudden death or arrhythmias (Diab, Poppenga, & Uzal, 2017; Kiryu et al., 1999; Kiryu, Nakamura, Kaneko, Oikawa, & Yoshihara, 1987; Lyle et al., 2011; Molesan et al., 2019). Only a few reports on conduction system pathology in nonathletic horses have been published, including cases that resembled arrythmogenic right ventricular cardiomyopathy involving myocardial loss and replacement by fatty or fibrous tissues (Else & Holmes, 1972; Raftery, Garcia, Thompson, & Sutton, 2015). In a study conducted in an abattoir on the cardiac lesions of old (˃ 10 years) nonathletic horses with clinical evidence of heart disease, approximately 94% of horses (17/18 cases) had microscopic myocardial lesions that primarily, included myocardial fibrosis (14/18 cases) and fatty metamorphosis of the myocardial fibres (7/18 cases) (Marcus & Ross, 1967). However, little else exists in terms of data on the cardiac pathologies of nonathletic horses (Buergelt, 2003; Buergelt & Del Piero, 2014; Else & Holmes, 1972; Marcus et al., 1967). Here we report on the gross and microscopic features of myocardial atrophy with fatty or fibrous tissues in a 13‐year‐old Arabian broodmare that had spontaneously died of suspected arrhythmia. During autopsy of the aged mare, we observed multifocal myocardial atrophy with concomitant fatty or fibrous tissues involving the conduction system; however, no coexistent signs of vascular pathology, myocardial infarction or myocardial inflammation were found. To the best of our knowledge, this is the first description of multifocal atrophic myocardial lesions involving the cardiac conduction system of non‐athletic horses.

## 2 CASE HISTORY

In March 2019, a groomer discovered a 13‐year‐old Arabian broodmare writhing in the sand paddock of a ranch. It had no history of health issues or cardiac symptoms. The ranch ʼs referral veterinarian later determined that the horse had died approximately 20 min after collapsing in the paddock. Electrocardiogram (ECG) before death detected a cardiac arrhythmia consistent with ventricular bigeminy. Three months before the incident, an endometrial biopsy and cytology of the uterine lavage fluid was performed. Results of a histopathological examination of the uterus corresponded to Grade 1, per the classification system proposed by Kenny & Doig (1986); no pathological abnormalities were observed in the endometrial tissues, including the vascular system and lymphatic vessels.

## 3 RESULT

### 3.1 Macroscopic changes

**FIGURE 1:**
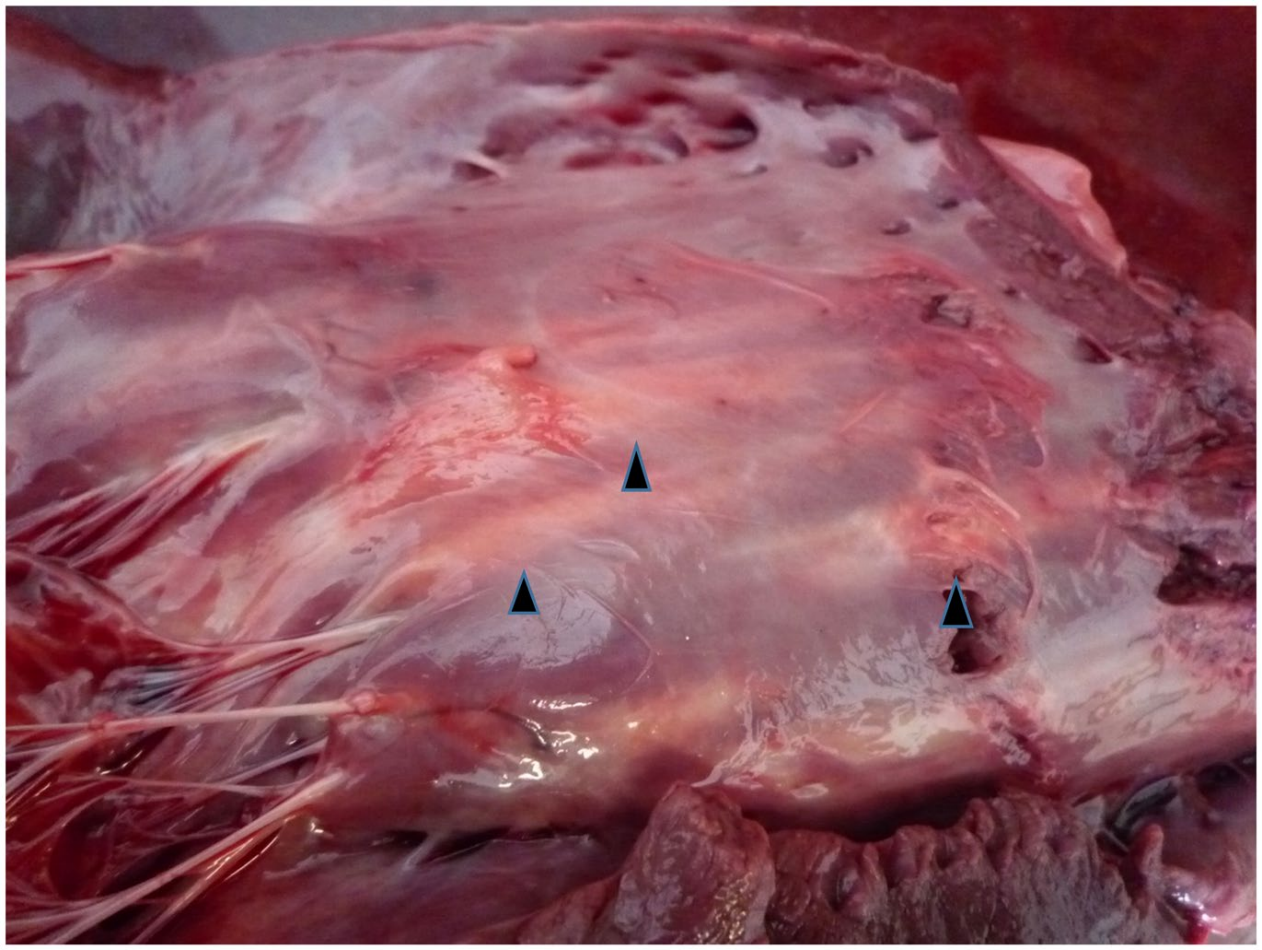
Gross photograph of the heart. Streaky and light greyish‐white lesions (arrowheads) were seen beneath the endocardium of the right ventricular free wall prior to formalin‐fixation

**FIGURE 2:**
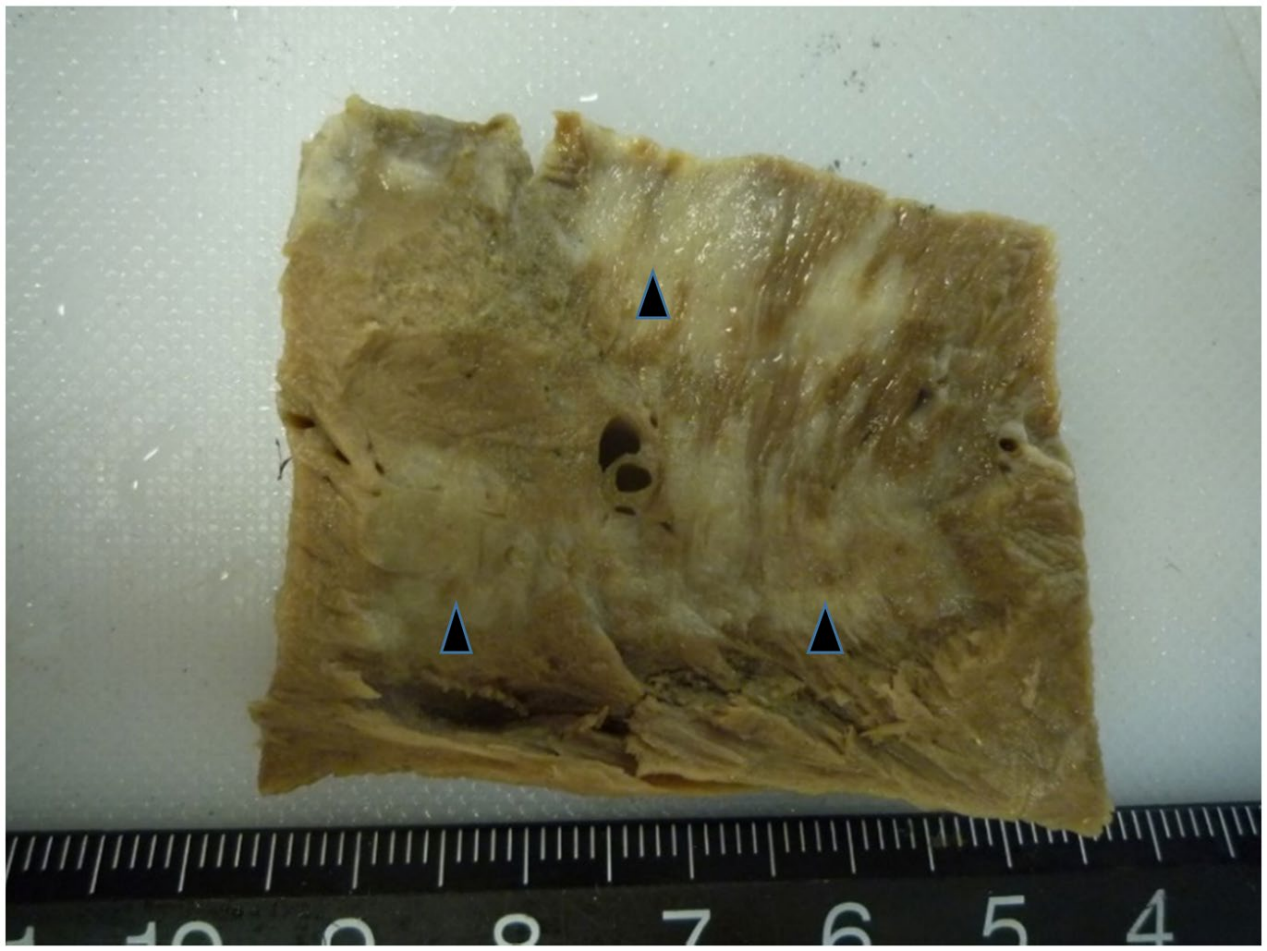
Gross photograph of the heart. Streaky and light greyish‐white lesions (arrowheads) were evident in the cross section of the muscular layers of the interventricular septum after formalin‐fixation

An autopsy was performed approximately 5 hr after death. Results of an external examination indicated a high degree of obesity; the subcutaneous and retroperitoneal tissues held large deposits of yellow fat. The atria and ventricles were dilated and filled with abundant dark‐red, poorly coagulated blood clots, and abundant fatty tissues were present outside the pericardial and epicardial surfaces in the right and left ventricles around the coronary blood vessels. The ventricles had conspicuous, sharply demarcated, unencapsulated, streaky, light greyish‐white lesions that varied in size but were generally massive, measuring 0.5– 8.0 x 1.0–15. 0 cm. They were isolated, regional (localized) or a coalescence of multiple lesions that were primarily located in the muscular layers of the right and left ventricular free walls (Figure 1) and interventricular septum (Figure 2). However, the greyish - white focal lesions within the myocardial walls did not appear to have been caused by the infiltration of epicardial or sub - endocardial adipose tissues. Gross greyish - white lesions in the myocardial muscular layer penetrated the endocardium and epicardium. Furthermore, gross grey to white muscular lesions were visible on the endocardial and epicardial surfaces of the heart.

In the interventricular septum, the lesions were distributed from the apex (basal superior wall) to the middle portion of the heart (middle superior wall). In the right and left ventricular free walls, the lesions were distributed from the inferior to the middle portion of the ventricular free walls, including the walls of the coronary artery. No gross lesions were found in the apex, atria, sinoatrial nodal region or atrioventricular nodal region. We did not observe other gross cardiac lesions, such as coronary aneurysm, medial degeneration of the aorta or extra‐pulmonary artery, or nodular fibrous plaques (associated with the migrating larvae of *Strongylus vulgaris*), in the intima of the aorta. However, there were gross changes in organs other than the heart, such as diffuse, mild to moderate pulmonary congestion; atelectasis; and congestion in the major internal organs and pia matter of the cerebrum and cerebellum.

### 3.2 Microscopic features

Based on a protocol developed by Diab et al. (2017), tissue samples of cardiac lesions were collected from the atrioventricular nodal region, sinoatrial nodal region, right and left atria, interventricular septum and the right and left ventricular free walls (Diab et al., 2017). Histopathological examination was performed by board certified veterinary pathologists (MGA and MO), who used a conventional method to examine 5 - *µ*m - thick sample slices that had been stained with either Masson ʼ s trichrome or haematoxylin and eosin.

**FIGURE 3:**
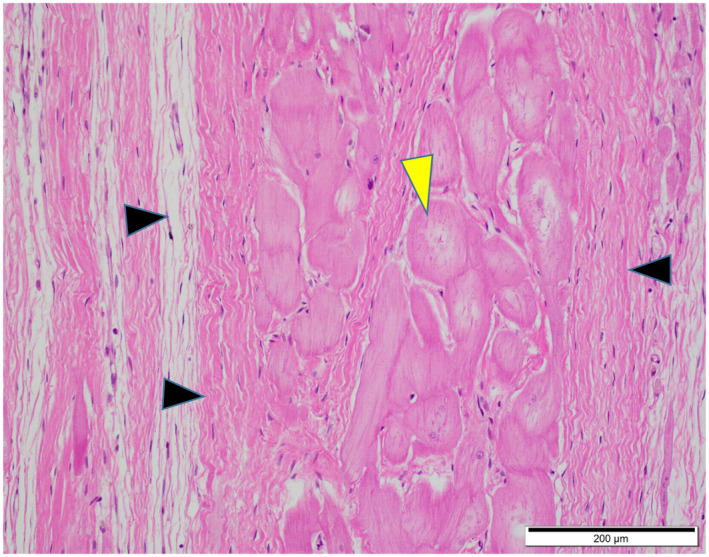
Photomicrograph of the heart. Marked atrophy and loss of the myocardial fibres (black arrowheads) around Purkinje fibres (yellow arrowhead). Haematoxylin and eosin stain. Scale bar = 200 µm

**FIGURE 4:**
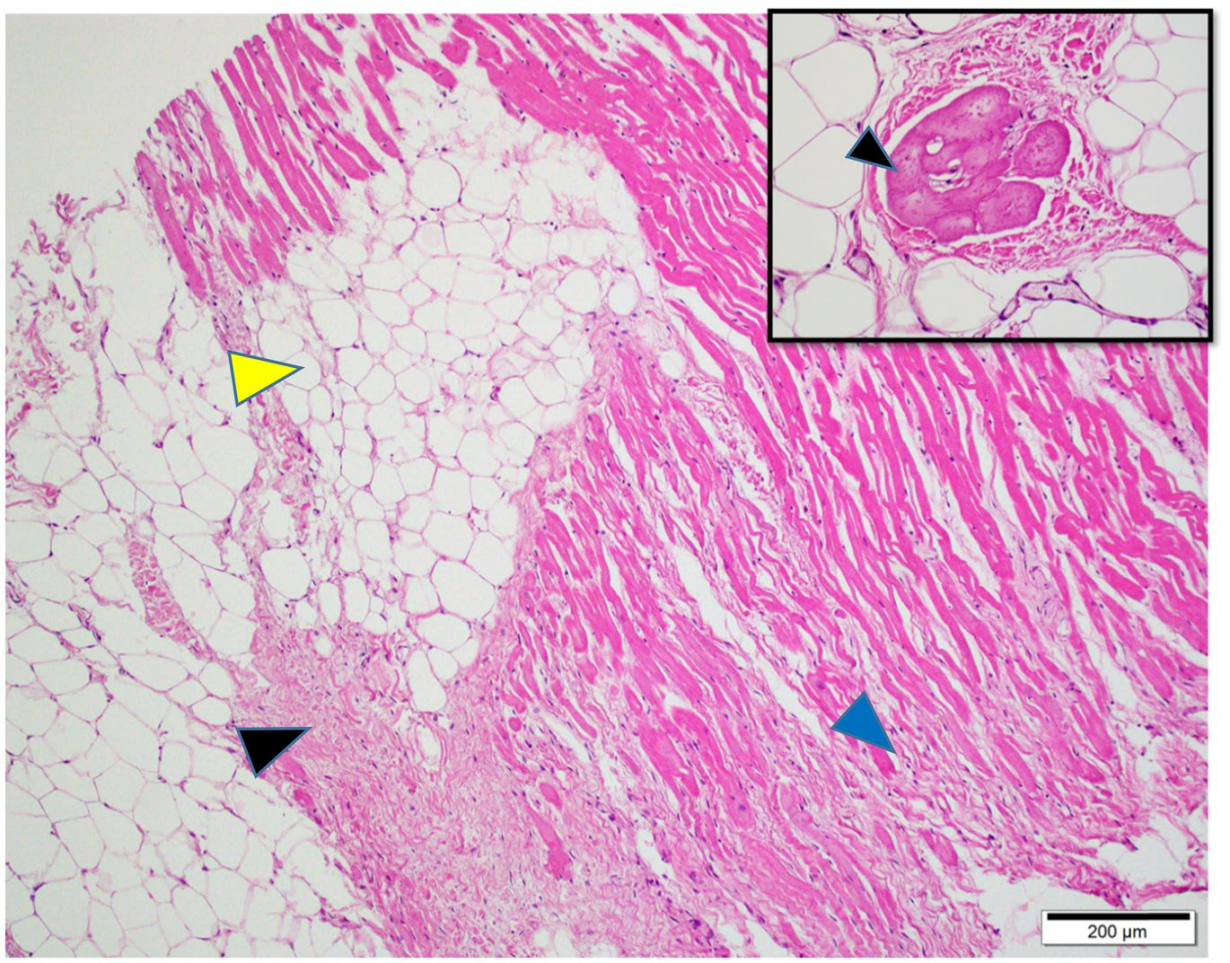
Photomicrograph of the heart. Myocardial interstitial fibrosis (black arrowhead), non‐encapsulated interstitial fat infiltrates (yellow arrowhead), and atrophy of the myocardial fibres (blue arrowhead). Inset indicates Purkinje fibres isolated within adipose tissue. Haematoxylin and eosin stain. Scale bar = 200 µm

**FIGURE 5:**
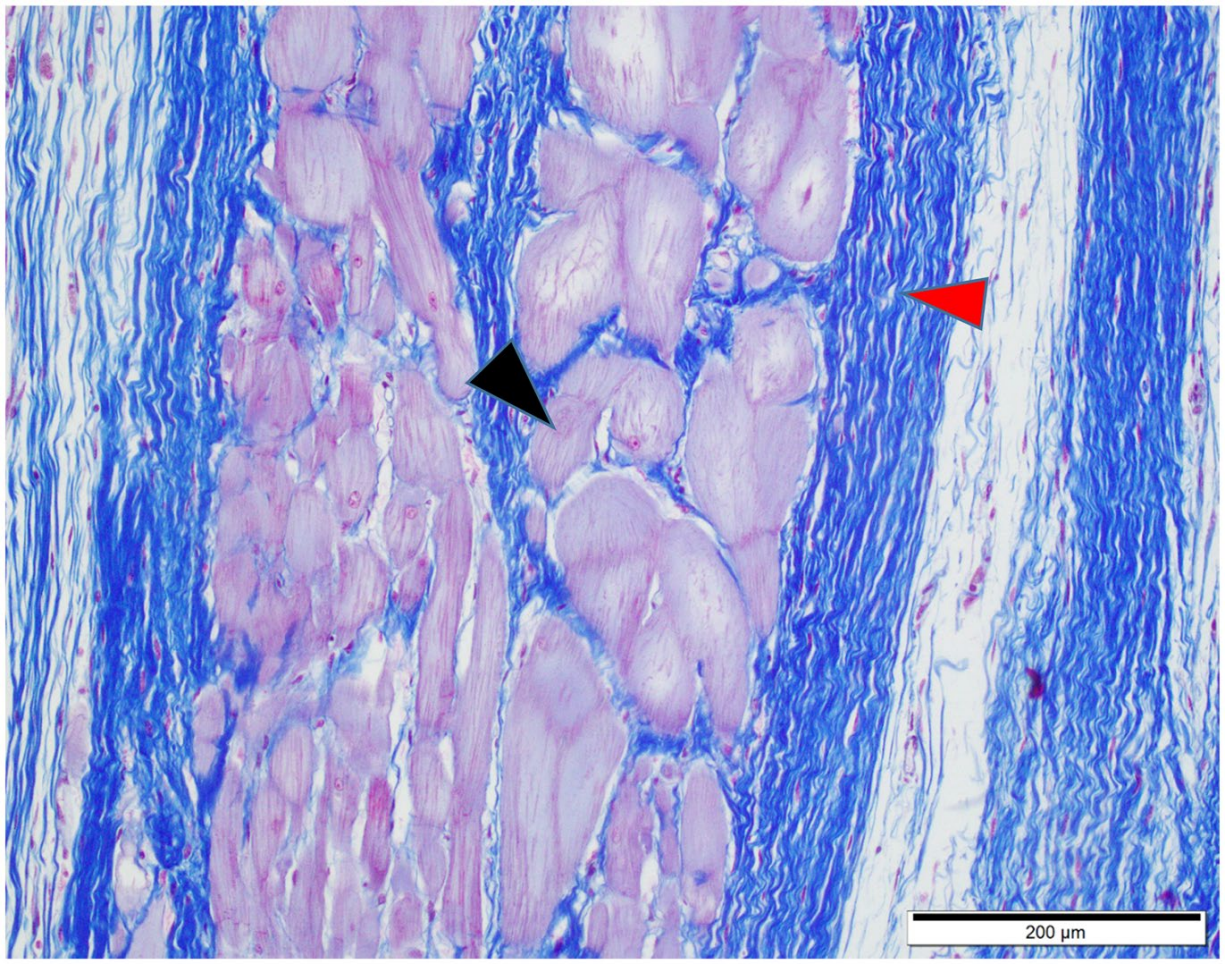
Photomicrograph of the heart. Purkinje fibres (black arrowhead) surrounded with collagen fibre (red arrowhead) stained in blue. Masson ʼ s trichrome staining. Scale bar = 200 µm

The macroscopically observed greyish‐white lesions of the ventricles were histologically correlated to myocardial atrophy with concomitant interstitial fibrosis and infiltration of adipose tissues, around the bundle of His (Figures 3, 4). The Purkinje fibres in the interventricular septum and right ventricular free walls were surrounded by a large amount of fibrous connective tissues adjacent to areas with myocardial atrophy (Figure 5) and were occasionally isolated within adipose tissues (Figure 4, inset). Within the interstitial fibrotic areas, perivascular infiltrates of lymphocytes and macrophages were minimal. There was no histologic evidence of ongoing myocardial degeneration and disintegration due to contraction necrosis, intense inflammation, or fatty infiltration into the cytoplasm of myocytes (i.e. lipid vacuolation of the sarcoplasm). There were no significant changes in nodal muscle cells of the sinoatrial or atrioventricular nodes, other than myocardial atrophy with interstitial fibrosis. There was no evidence of myocardial atrophy - induced pathologies, such as infarct, ischaemic lesions, myocardial degeneration, myocarditis and endocarditis, and no evidence of major vascular pathologies, such as atherosclerosis, arteriosclerosis and medial degeneration in myocardial tissues. No lesions were observed in the skeletal muscles that would have suggested muscular dystrophies, and no haemosiderin - laden macrophages in the pulmonary alveoli that would have suggested chronic congestive heart failure.

## 4 DISCUSSION

The cardiac lesions in the present case were histologically characterized by multifocal myocardial atrophy with concomitant fatty infiltration and myocardial fibrosis between cardiomuscular fibres/bundles, and nests of Purkinje fibres that were sequestered primarily by prominent fibrous connective tissues or adipose tissues in the interventricular septum and the free walls of both ventricles.

In animals, the atrophy of cardiac myocytes develops secondarily to conditions such as cachexia, myocarditis, ischaemia due to coronary arteriosclerosis, tumour compression, muscular dystrophy or hereditary loss of cardiomyocytes (Agudelo, Svoboda, Husnik, & Dvir, 2013; Buergelt, 2003; Miller & Gal, 2017). However, the aetiology of atrophy in the present case was not discernible in the histologic sections examined. Atrophic myocytes were multifocally distributed near intact myocytes and adjacent to areas of fatty infiltration and/or myocardial fibrosis, but such features were not found beneath well‐developed adipose tissues in the epicardium. Based on the lack of mass formation, anatomic location and degree of fatty infiltration, the accumulation of adipocytes was considered to be a pathologic secondary process that resulted from atrophy/loss of cardiomyocytes, rather than a primary neoplastic proliferation of adipocytes (lipoma). Thus, while it is possible that the release of free fatty acids from adipose tissues had induced myocardial toxicity that resulted in the apoptosis of cardiac myocytes, the possibility that pressure from fat accumulation or the compressive growth of adipocytes had induced myocardial atrophy can be excluded (Agudelo et al., 2013).

Myocardial fibrosis has been reported in old horses above 12 years in association with areas of myocardial degeneration and arteriosclerotic changes (Marcus & Ross, 1967), and in horses affected with aortic strongylosis (Cranley & McCullagh, 1981). In the present case, no significant aortic strongylosis or vascular changes, such as arteriosclerosis, atherosclerosis and medial degeneration were observed. With regard to morphogenesis, myocardial fibrosis appeared to have originated from interstitial connective tissues, from which it spread between atrophic cardiac myocytes.

Cases similar to ours have been reported, in which nonathletic horses suffered cardiac fatty changes (Baker & Kreeger, 1987; Freel, Morrison, Thompson, & Else, 2010; Hamir, Habecker, & Tulleners, 1994; Raftery et al., 2015) and Purkinje fibres surrounded by adipose tissue (Marcus & Ross, 1967). Two of the five reports had discussed fibrofatty infiltration of the myocardium as a possible cause of sudden cardiac death (Freel Raftery et al., 2015). However, no mention was made of myocardial atrophy. Thus, the pathogenesis of myocardial atrophy remains obscure in the present case. To the best of our knowledge, this is the first report to provide a histopathological description of multifocal myocardial atrophy involving the Purkinje fibres of an Arabian broodmare that had suffered sudden death.

## CONFLICTS OF INTEREST

The authors declared no potential conflicts of interest with respect to the research, authorship and/or publication of this article.

## AUTHOR CONTRIBUTION

**Midori Goto Asakawa and Masa‐aki Oikawa:** Conceptualization; Formal analysis; Investigation; Validation; Writing‐original draft; Writing‐review & editing. **Wasiq Mehmood:** Formal analysis; Investigation; Methodology; Validation; Writing‐review & editing. **Mohammed Ali:** Conceptualization; Investigation; Project administration; Resources; Supervision; Validation; Writing‐review & editing.

## ETHICAL STATEMENT

The authors confirm that the ethical policies of the journal. As noted on the journal`s author guidelines page, have been adhered to. No ethical approval was required as this is an investigation of an animal at post‐mortem examination.

### PEER REVIEW

The peer review history for this article is available at https://publons.com/publon/10.1002/vms3.350.
